# Pharmacokinetic comparison of sitagliptin and metformin HCl extended-release tablets versus JANUMET^®^ XR in healthy volunteers under fasting and fed conditions

**DOI:** 10.3389/fphar.2023.1105767

**Published:** 2023-03-22

**Authors:** Linling Que, Wei Qin, Yunfei Shi, Ying Ding, Kai Huang, Zhenzhong Qian, Bingjie Huang, Peipei Zhou, Qing He

**Affiliations:** ^1^ Drug Clinical Trial Institution, Wuxi People’s Hospital Affiliated to Nanjing Medical University, Wuxi, China; ^2^ Nanjing Chia-Tai Tianqing Pharmaceutical Company, Nanjing, China

**Keywords:** sitagliptin, metformin, Janumet^®^ XR, diabetes, bioequivalence, pharmacokinetics

## Abstract

**Background and Objectives:** Janumet^®^ XR is the combination of sitagliptin and extended metformin hydrochloride produced by Merck Sharp & Dohme. It is specially designed for diabetes mellitus patients taking both drugs already. Janumet^®^ XR exhibited clinically significant blood glucose lowering efficacy and long-term use safety. However, no generic form of Janumet^®^ XR has been approved in western countries. The relatively high cost made the medication less prescribed. A more affordable form of this drug may benefit an immense diabetes mellitus population. The current study compared the bioequivalence (BE) of sitagliptin 100 mg and metformin 1000 mg produced by Nanjing Chia-Tai Tianqing Pharmaceutical Company to Janumet^®^ XR in healthy Chinese subjects.

**Methods:** Twenty-eight healthy Chinese subjects were enrolled in Study 1 and 2, respectively. Both studies were conducted with an open, randomized, two-period crossover design using the test (T) or the reference (R) drug. Study 1 is conducted under the fasting state, and Study 2 is under the fed state. Subjects received an oral dose of sitagliptin 100 mg and metformin 1000 mg, and plasma concentrations of sitagliptin and metformin were determined up to 72 h post-dose. Pharmacokinetic (PK) parameters, including maximum serum concentration (C_max_) and area under the concentration-time curve up to the last quantifiable concentration (AUC_0–t_) of both sitagliptin and metformin, were calculated and compared between the T and R treatments.

**Results:** In the fasting study, the geometric mean ratios of C_max_, AUC_0–t_, and AUC_0-∞_ for sitagliptin were 109.42%, 101.93%, and 101.95%, respectively; the corresponding ratios for metformin were 98.69%, 94.12%, and 93.42%, respectively. In the fed study, the geometric mean ratios of C_max_, AUC_0–t_, and AUC_0-∞_ for sitagliptin were 98.41%, 100.30%, and 100.24%, respectively; the corresponding ratios for metformin were 97.79%, 99.28%, and 100.69%, respectively. The 90% CIs of C_max_, AUC_0–t_, and AUC_0-∞_ in both studies were all within acceptance limits (80.00%–125.00%).

**Conclusion**: The results demonstrated for the first time that sitagliptin 100 mg and metformin 1000 mg produced by Nanjing Chia-Tai Tianqing Pharmaceutical Company was bioequivalent to the branded Janumet^®^ XR, and both drugs were well tolerated.

## 1 Introduction

Type 2 diabetes mellitus (T2DM) patients often require a multi-drug regimen to achieve adequate glycaemic control. Janumet^®^ is a combination of two active substances, sitagliptin and metformin hydrochloride. Sitagliptin is a selective inhibitor of dipeptidylpeptidase-4, thus slowing the rapid inactivation of incretins, which helps lowering postprandial glucose (Lyseng-Williamson, 2007). Metformin is a biguanide compound used as the first-line drug in T2DM treatment for its well-established safety and effectiveness in lowering blood glucose (Flory and Lipska, 2019). The use of sitagliptin in combination with metformin consistently demonstrated a clinically significant reduction in average glycosylated hemoglobin than metformin monotherapy in inadequately controlled T2DM patients (Charbonnel et al., 2006; Goldstein et al., 2007; Nauck et al., 2007; Scheen et al., 2010; Arechavaleta et al., 2011; Schernthaner et al., 2013). In addition, the bioequivalence of Janumet^®^ *versus* equivalent doses of sitagliptin and metformin as individual tablets was demonstrated in healthy subjects (Janumet, 2022). Janumet^®^ was approved by the United States of America (US) Food and Drug Administration (FDA) in 2007 as an adjunct to diet and exercise to improve glycemic control in T2DM patients who were not adequately controlled on metformin or sitagliptin alone or in patients already being treated with both drugs (Janumet, 2022).

Janumet^®^ was supposed to be given twice daily. To bring the convenience of once-daily dosing, FDA approved Janumet^®^ XR designed for a fixed dosing regimen of sitagliptin and extended-release metformin in February 2012. It was available as 100 mg/1000 mg, 50 mg/1000 mg, and 50 mg/500 mg. Compared with the immediate release form, the extended form of metformin exhibits steadier glycemic control, better gastrointestinal tolerance, and improves patient adherence. Nevertheless, the method of developing extended-release metformin core tablet and synergistic coating of sitagliptin immediate-release (IR) formulation requires a more sophisticated formula and preparation technique (Ahmed et al., 2022a). Up to now, no generic products of Janumet^®^ XR have been authorized by FDA or European Medicines and Healthcare Products Regulatory Agency (MHRA).

FDA released a draft guidance on Metformin Hydrochloride; Sitagliptin Phosphate (Tablet, Extended Release) in July 2014 (US Food and Drug Administration, 2014). Both fasting and fed studies are suggested. Single-dose, two-way crossover design is recommended. The drug should be administered with 240 ml of a 20% glucose solution in water, followed by 60 ml of the same solution administered every 15 min for up to 4 h after dosing. This dosing method was recommended mainly to avoid hypoglycemia. The drug strength studied was 1000 mg/EQ 100 mg base. Other strengths can gain waivers of *in vivo* testing under three conditions, including bioequivalence of 1000 mg/EQ 100 mg base strength, acceptable *in-vitro* dissolution testing of all strengths, and proportional similarity of the formulations across all strengths.

The current study was conducted under the FDA guidance to compare the bioequivalence (BE) of sitagliptin 100 mg and metformin 1000 mg produced by Nanjing Chia-Tai Tianqing Pharmaceutical Company to Janumet^®^ XR. This was the first reported bioequivalent study of Janumet^®^ XR, and the study may shed light on the clinical study design and conduction of Janumet^®^ XR BE.

## 2 Methods

### 2.1 Study designs and treatments

There were two studies in the project. Study 1 was under the fasting state, and study 2 was under the fed state. Both studies were conducted with an open, randomized, two-period crossover design using the T or R drug at the dose of sitagliptin 100 mg and metformin 1000 mg. The studies aimed to compare the PK profiles and safeties of the T and R drugs. The study protocols were approved by the Wuxi People’s Hospital Ethics Committee (Approval No. 2018LLPJ-I-14-03) and were conducted at Wuxi People’s Hospital Affiliated to Nanjing Medical University, Jiangsu Province, China. The studies were performed in accordance with the principles of the World Medical Association (WMA) Declaration of Helsinki. All volunteers signed written informed consents before enrollment.

The R drug was JANUMET^®^ XR (sitagliptin 100 mg and metformin 1000 mg HCl extended-release) tablets produced by Merck Sharp & Dohme Corp, the United States, batch number R005485; The T drug was sitagliptin 100 mg and metformin 1000 mg HCl extended-release tablets produced by Nanjing Chia-Tai Tianqing Pharmaceutical Company, batch number 180503.

**FIGURE 1:**
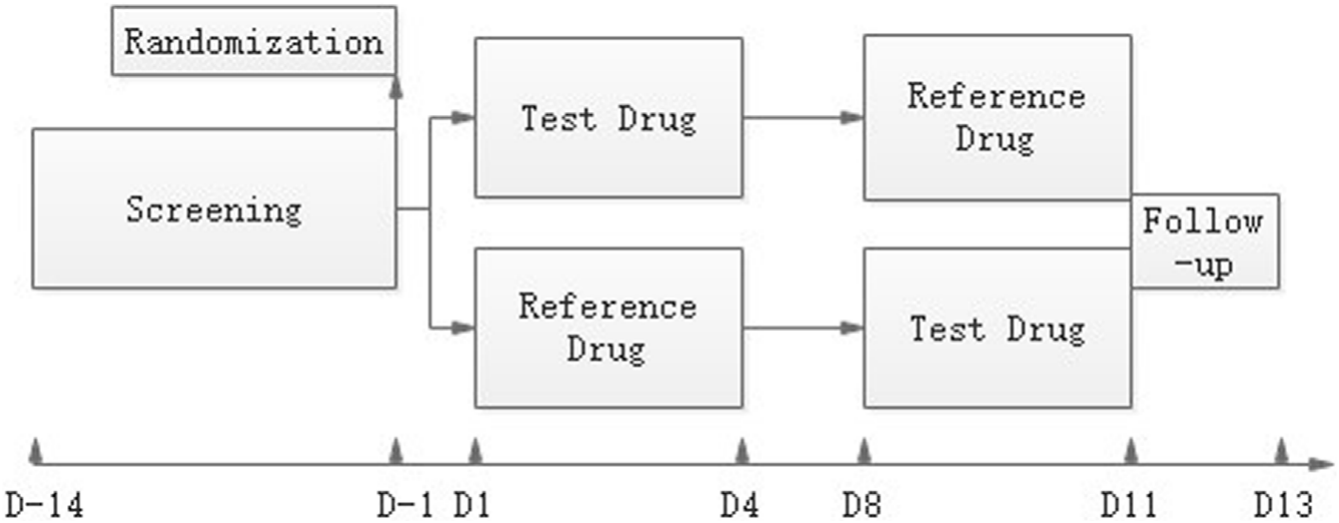
Study design. After a maximum screening of 14 days, eligible subjects were randomized on day-1. The reference drug JANUMET^®^ XR (sitagliptin 100 mg and metformin 1000 mg HCl extended-release) tablets or the test drug was given on day 1 or day 8 in a randomized R-T or T-R sequence, and serial PK samples were collected. A follow-up visit was done on day11-13.

Subjects received a single oral dose of the T or R drug in each period in a randomized way under either fasting or fed conditions, with a wash-out of 7 days. The study design is shown in Figure 1. For the fed study, subjects finished the high-fat diet (containing protein 16.52%, fat 56.85%, carbohydrate 26.63%) in 30 min and were given the drug with 240 ml of a 20% glucose solution in water 30 min after they started eating the diet. For the fasting study, the drug was administered with 240 ml of a 20% glucose solution in water under the fasting state. An overnight fast for at least 10 h before administration was required. Subjects were not allowed to drink water 1 h before and after dosing, and lunch could be served only 4 h after dosing for both studies.

### 2.2 Subjects

Healthy men and women above 18 years with a body mass index of 19–26 kg/m^2^ were enrolled. Bodyweight was required ≧ 50 kg for men and ≧ 45 kg for women. Main exclusion criteria included: the presence of any disease; Physical examination, blood test (hematology, serum biochemistry and prothrombin time), and urinalysis of clinical significance; Unwillingness to take contraception measures during the study period and in 3 months after the end of the study; Blood donation or blood loss of more than 400 ml 3 months prior to screening; had surgery 3 months prior to screening and during the study period; Creatinine clearance rate (CrCl) < 80 ml/min; fasting plasma glucose ≦ 3.9 mmol/L or ≧ 6.1 mmol/L; Allergic to sitagliptin or metformin; Positive results of human immunodeficiency virus (HIV), hepatitis B surface antigen (HBsAg), hepatitis C virus (HCV), and TP (*Treponema pallidum*) test; Positive results of nicotine, alcohol, and drugs; Take any medication 14 days prior screening; Pregnant women or positive results of human chorionic gonadotropin (HCG) test; Participation in any clinical trial during the 3 months prior to screening.

### 2.3 Blood sampling and bioanalytical assay

Venous blood samples (4 ml) were collected from an intravenous indwelling catheter before dosing and at 0.5, 1, 1.5, 2, 2.5, 3, 3.5, 4, 4.5, 5, 6, 7, 8, 9, 10, 11, 12, 14, 24, 32, 48, and 72 h after dosing for both fed and fasting studies. Blood samples were drawn into BD Vacutainers^®^ with ethylene diamine tetra acetic acid (EDTA) anticoagulant. Plasma was separated by centrifugation at 2150 ± 5 g for 10 min at 2°C–8°C and stored at −20°C within 2 h after sampling and transferred to −80°C within 24 h.

Plasma concentrations of sitagliptin and metformin were analyzed using a fully validated liquid chromatography-tandem mass spectrometry (LC-MS/MS) method. The calibration curves for sitagliptin and metformin were 1.0–700 ng/ml and 4.0–2800 ng/ml, respectively. In the fed study, the mean intra-batch precision and accuracy of this method were 2.4%–3.8% and 96.2%–102.5%, respectively, for sitagliptin and 2.5%–3.8% and 98.1%–104.6%, respectively, for metformin. In the fasting study, the mean intra-batch precision and accuracy of this method were 2.9%–4.5% and 97.0%–102.7%, respectively, for sitagliptin and 2.8%–3.5% and 99.5%–104.2%, respectively, for metformin.

### 2.4 Pharmacokinetic parameters

Serum sitagliptin and metformin pharmacokinetic parameters C_max_ (maximum serum concentration), T_max_ (time to achieve C_max_), AUC_0-t_ (area under the concentration-time curve from time 0 to the last quantifiable concentration time t), AUC_0–∞_ (AUC extrapolated to infinity) and half-life (t_½_) were determined or calculated with the validated software Phoenix WinNonLin^®^8.1, Certara, Inc., using a non-compartmental analysis and the linear trapezoidal rule.

### 2.5 Safety

Vital signs (blood pressure, pulse, temperature, breath) were assessed during each treatment period pre-dose (0 h) and post-dose at 2, 8, 24, 48, and 72 h. Finger point blood glucose was measured 2 and 8 h after dosing. Adverse events (AEs) were recorded continuously throughout both studies according to National Cancer Institute Common Terminology Criteria Adverse Events (NCI CTCAE, Version 5.0. The clinical significance of abnormal results was determined by physicians. If clinical abnormalities were present, follow-ups were required.

### 2.6 Sample size

Based on the results of sitagliptin dispensatory Januvia^®^, the within-subject coefficient of variations (CV) for the area under the plasma-concentration time curve AUC was 5.8%, and the CV for maximum plasma concentration (C_max_) was not reported (Januvia, 2006). The CV for the PK parameters (C_max_, AUC) was assumed to be 20% for metformin immediate release (IR) based on previous studies (Park et al., 2015; El Messaoudi et al., 2016), and the CV was reported equivalent between extended released (XR) and IR metformin (Timmins et al., 2005). To achieve a statistical power of 80% that a two-sided 90% confidence interval (CI) for the ratio of PK parameters (C_max_, AUC) between two treatments would be contained within the 0.80–1.25 limit, the study required 19 evaluable subjects in the fasting and fed studies, respectively. Twenty-eight subjects were finally enrolled in case of withdrawals, respectively.

### 2.7 Statistical analyses

Study data were summarized by descriptive statistics. The statistical analyses were performed using SAS^®^ version 9.4. For the comparisons, C_max_, AUC_0–t_, and AUC_0–∞_ were analyzed using linear mixed models after logarithmic transformation. Bioavailability was achieved if the 90% CI of each parameter geometric means ratio (GMR) test/reference fell between 0.8–1.25. The statistical analysis included treatment, period, sequence, and subject-within-sequence as fixed effects. T_max_ was compared between treatments using the non-parametric Wilcoxon signed-rank test.

## 3 Results

### 3.1 Subjects

Twenty-eight healthy men and women were randomized and completed the fasting study, and all were included in the safety evaluation and pharmacokinetic analysis. Twenty-eight healthy men and women were randomized in the fed study. One subject quit the study for personal affairs after finishing the first period, and the other 27 subjects completed the whole study. All the subjects were included in the safety evaluation and the pharmacokinetic analysis.

The baseline demographics of the participants are detailed in Table 1.

**TABLE 1:** Baseline demographics of study participants.

Characteristics	Fasting Study (n=28)		Fed Study (n=28)	
	T-R(n=14)	R-T(n=14)	T-R	R-T
Age(Years)	30.1±7.5	31.1±6.2	28.9±7.9	26.3±4.8
Sex, n(%)				
Male	6 (42.9)	10 (71.4)	10(71.4)	11(78.6)
Female	8 (57.1)	4 (28.6)	4(28.6)	3(21.4)
Height(cm)	161.0±6.2	166.6±9.4	164.7±8.4	167.9±7.4
Weight(kg)	60.4±7.3	62.7±9.4	62.4±9.3	63.0±6.5
BMI(kg/m^2^)	23.2±1.8	22.5±1.9	22.9±2.3	22.4±1.8

Data are the mean ± SD, except for sex (male/female), which is the %; BMI, body mass index; SD, standard deviation. a% = n/N*100.

### 3.2 Pharmacokinetics

#### 3.2.1 Fasting study

**FIGURE 2:**
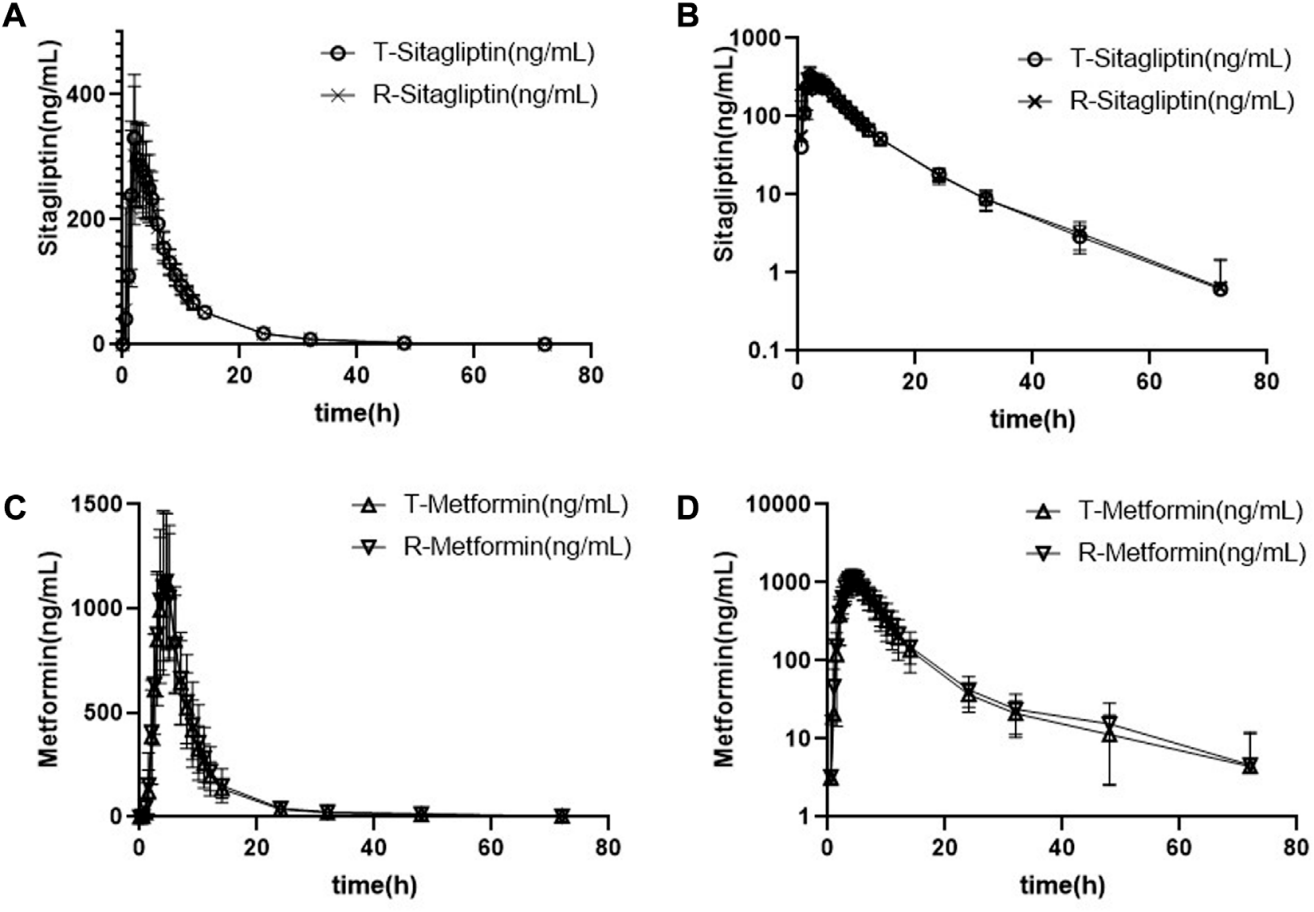
Mean plasma sitagliptin and metformin concentration-time profiles after dosing under the fasting state. Error bars represent the standard deviations. **(A)** Sitagliptin, linear scale, **(B)** sitagliptin, semi-log scale, **(C)** metformin, linear scale, and **(D)** metformin, semi-log scale.

The mean plasma sitagliptin and metformin concentration-time profiles under the fasting state are shown in Figure 2. The curves of both sitagliptin and metformin were very similar between the T and R drugs. Sitagliptin level after the T drug manifested a slightly higher mean C_max_ value and a shorter T_max_ than the R drug. The mean C_max_ and T_max_ values for metformin were very close between the T and R drugs. PK parameters of the two groups are summarized in Table 2. BE evaluation of the two groups is shown in Table 3. The geometric mean ratios (T/R) of C_max_, AUC_0–t_, and AUC_0-∞_ for sitagliptin were 109.42%, 101.93%, and 101.95%, respectively, the corresponding ratios for metformin were 98.69%, 94.12%, and 93.42%, respectively. The 90% CIs were all within acceptance limits (80.00%–125.00%).

**TABLE 2:** Pharmacokinetic parameters after single-dose administration of test and reference drug under fasting state.

Parameters	Sitagliptin		Metformin	
	Test Drug	Reference Drug	Test Drug	Reference Drug
C_max_/ng/mL	378.67±91.79	346.15±89.97	1211.46±372.69	1212.50±316.97
AUC_0-t_/h*ng/mL	2700.89±366.12	2665.70±330.04	8327.22±2174.41	8796.45±2255.33
AUC_0-∞_/h*ng/mL	2729.81±366.81	2693.87±334.48	8593.84±2348.91	9119.98±2325.40
T_max_/h	2.00(1∼5)	2.50(1.5∼5)	4.50(3∼6)	4.50(3∼8)
T_1/2z_/h	9.60±2.97	10.08±2.58	14.11±11.77	16.17±12.33
λz/1/h	0.08±0.02	0.07±0.02	0.08±0.05	0.07±0.05

Data are the mean ± SD, except T_max_ are median (min, max).

**TABLE 3:** Statistical comparison of Sitagliptin and Metformin Pharmacokinetic parameters under fasting state.

Parameters		Test Drug	Reference Drug n=27	Ratio% (T/R)	90% CI	Intra-individual variation %
		N=28				
C_max_/ng/mL	Sitagliptin	366.64	335.06	109.42	90.40∼107.73	19.42
	Metformin	1155.41	1170.79	98.69	92.67∼103.18	11.61
AUC_0-t_/h*ng/mL	Sitagliptin	2674.81	2624.04	101.93	99.01∼104.95	6.05
	Metformin	8021.92	8522.66	94.12	88.72∼99.85	13.01
AUC_0-∞_/h*ng/mL	Sitagliptin	2704.44	2652.70	101.95	99.03∼104.96	6.04
	Metformin	8256.81	8838.60	93.42	87.94∼99.23	13.31

Data are the mean ± SD.

#### 3.2.2 Fed study

**FIGURE 3:**
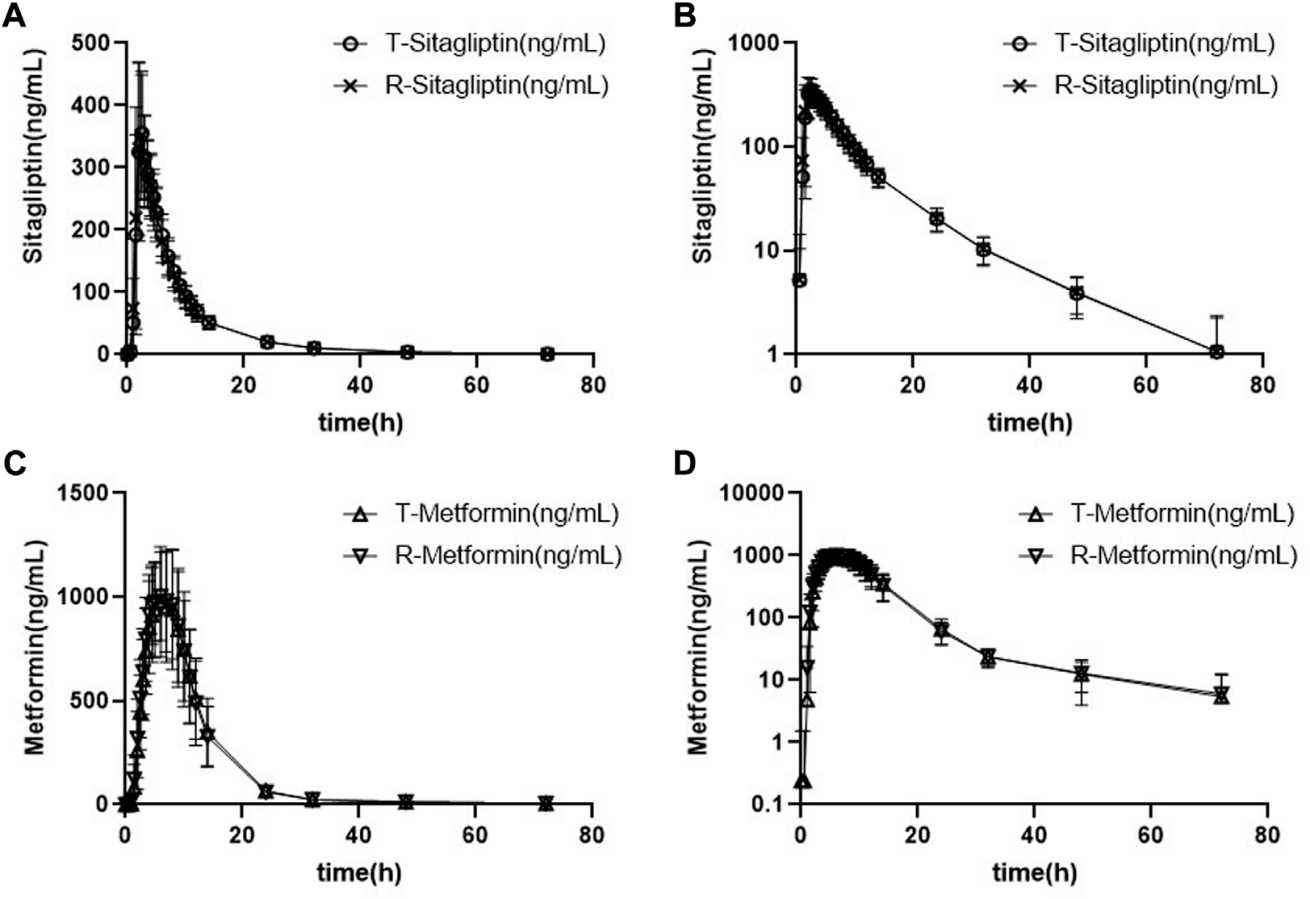
Mean plasma sitagliptin and metformin concentration-time profiles after dosing under the fed state. Error bars represent the standard deviations. **(A)** Sitagliptin, linear scale, **(B)** sitagliptin, semi-log scale, **(C)** metformin, linear scale, and **(D)** metformin, semi-log scale.

The mean plasma sitagliptin and metformin concentration-time profiles under the fed state are shown in Figure 3. The curves of both sitagliptin and metformin were very similar between the T and R drugs. The mean C_max_ value after the R drug was slightly higher than the T drug for both sitagliptin and metformin. The mean T_max_ value was very close between the T and R drugs for both sitagliptin and metformin. Compared to the fasting study, both C_max_ and AUC_0–t_ of sitagliptin increased under the fed state. While the mean C_max_ and AUC_0–t_ of metformin decreased and T_max_ extended under the fed state compared to the fasting state. PK parameters of the two groups are summarized in Table 4. BE evaluation of the two groups is shown in Table 5. The geometric mean ratios (T/R) of C_max_, AUC_0–t_, and AUC_0-∞_ for sitagliptin were 98.41%, 100.30%, and 100.24%, respectively, the corresponding ratios for metformin were 97.79%, 99.28%, and 100.69%, respectively. The 90% CIs were all within acceptance limits (80.00%–125.00%).

**TABLE 4:** Pharmacokinetic parameters after single-dose administration of test and reference drug under fed state.

Parameters	Sitagliptin		Metformin	
	Test Drug	Reference Drug	Test Drug	Reference Drug
C_max_/ng/mL	405.93±107.27	410.89±89.67	1050.71±235.26	1077.89±228.49
AUC_0-t_/h*ng/mL	2759.11±363.14	2749.29±378.76	11729.38±3342.83	11854.14±3091.46
AUC_0-∞_/h*ng/mL	2799.03±371.07	2790.03±384.11	12031.08±3303.73	12023.27±3128.63
T_max_/h	2.25(1∼4.5)	2.00(1∼3.5)	6.00(3.5∼9)	6.00(4∼10)
T_1/2z_/h	11.11±3.66	11.78±3.57	16.62±29.78	11.35±7.98
λz/1/h	0.07±0.02	0.06±0.02	0.09±0.04	0.08±0.04

Data are the mean ± SD, except T_max_ are median (min, max).

**TABLE 5:** Statistical comparison of Sitagliptin and Metformin Pharmacokinetic parameters under fed state.

Parameters		Test Drug	Reference Drug n=27	Ratio% (T/R)	90% CI	Intra-individual variation %
		N=28				
C_max_/ng/mL	Sitagliptin	393.39	399.75	98.41	91.43∼105.92	15.9
	Metformin	1025.80	1049.02	97.79	92.67∼103.18	11.61
AUC_0-t_/h*ng/mL	Sitagliptin	2736.09	2728.01	100.30	97.70∼102.96	5.65
	Metformin	11310.06	11392.44	99.28	95.53∼103.17	8.28
AUC_0-∞_/h*ng/mL	Sitagliptin	2775.46	2768.92	100.24	97.60∼102.95	5.75
	Metformin	11636.43	11556.38	100.69	96.89∼104.64	8.29

Data are the mean ± SD.

### 3.3 Safety

In the fasting study, 5 out of 28 subjects (17.9%) experienced AEs. The AEs related to the T drug were hypoglycemia and elevation of γ-glutamyltransferase. One subject experienced transient hypoglycemia and recovered after taking 60 ml of a 20% glucose solution in water. The AEs related to the R drug were ECG ST segment depression, decreased leukocyte count and neutrophil count. Elevation of γ-glutamyltransferase ended with recovering, and the other AEs recovered.

In the fed study, 4 out of 28 subjects (14.3%) experienced AEs. No AEs related to the T drug were reported. The AEs related to the R drug were decreased leukocyte count and decreased neutrophil count. All AEs recovered before the study ended.

None of the subjects were withdrawn from the two studies because of AEs. The data are summarized in Table 6.

**TABLE 6:** Summary of the number and types of adverse events that occurred in fasting and fed studies.

Symptoms	Numbers (%)			
	Fasting Study		Fed Study	
	T(N=28)	R(N=28)	T(N=28)	R(N=27)
Elevation of γ- glutamyltransferase	1(3.6)	-	-	-
Decrease in leukocyte count	-	1(3.6)	-	1(3.7)
Urine leukocyte	-	1(3.6)	-	-
Urine protein	-	1(3.6)	1(3.6)	-
ECG ST segment depression	-	1(3.6)	-	-
Decrease in neutrophil count	-	1(3.6)	-	1(3.7)
Hypoglycemia	1(3.6)	-	-	-
Injection fear	-	1(3.6)	-	-
Epistaxis	-	-	1(3.6)	-
Sinus bradycardia	-	-	-	1(3.7)

## 4 Discussion

This study aimed to compare the pharmacokinetic profiles and safety of sitagliptin and metformin HCl extended-release tablets with JANUMET^®^ XR in healthy volunteers. The results supported the bioequivalence of the T drug to the R drug in terms of both rate (C_max_) and extent (AUC_0−t_) of absorption under fasting and fed conditions. The PK parameters were similar to the published FDA review data (FDA, 2005; FDA, 2006; FDA, 2012) under the fed state, with a slightly lower AUC_0−∞_ and C_max_ for metformin in our study and a slightly higher C_max_ and lower AUC_0−∞_ for sitagliptin results. Under the fasting state, the C_max_ of sitagliptin was consistent with the FDA review data, while AUC_0−∞_ was slightly lower in our study. The PK parameters of metformin after taking JANUMET^®^ XR under the fasting state were not available in the FDA review data. Recently, BE studies of sitagliptin (100 mg) and fixed-dose combination (FDC) of sitagliptin–metformin (50/1000 mg) IR have been reported (Schnaars et al., 2023). The PK parameters C_max_, AUC_0−t_, AUC_0−∞_ of sitagliptin (100 mg) under the fasting condition were approximately 14–32% higher than our data. The results indicated a combination formulation may decrease the absorption rate and extent of sitagliptin. Metformin (1000 mg, IR) exhibited a much higher C_max_ (1592.89 ng/L), shorter median T_max_ (3.5 h), and similar AUC results compared to our XR products under the fed condition. The results showed metformin (XR) extended the releasing process and exhibited steadier blood concentration than metformin (IR).

The absorption of sitagliptin in terms of C_max,_ AUC_0−t_ and AUC_0−∞_ was slightly higher under the fed state than the fasting state, which was opposite to the FDA review data that the AUC_0−∞_ and C_max_ for sitagliptin were slightly decreased under the fed state. On the contrary, the high-fat diet slightly decreased the absorption rate of metformin with a lower C_max_ and longer T_max_ while increasing the AUC_0−t_ by approximately 35% and AUC_0−∞_ by approximately 32% compared to the fasting state. It was generally consistent with the JANUMET^®^ XR FDA review data (FDA, 2012) that a high-fat breakfast decreased the C_max_ for metformin by approximately 9% and increased AUC_0−∞_ for metformin by approximately 62%. A BE study of Janumet reported a slightly higher C_max_ of sitagliptin under the fed state, and the AUC_0−t_ and AUC_0−∞_ were comparable between the fasting and fed condition (Shi et al., 2022). The results were consistent with ours. In summary, the intake of a meal within 30 min of dosing can limitedly affect the absorption extent of sitagliptin while increasing the absorption extent of metformin.

The general study design followed the FDA guidance as single-dose, two-way crossover. Two types including fasting and fed were conducted. Healthy men and non-pregnant women were included. Our study provided some details regarding the design of sampling point and hypoglycemia prevention. The T_max_ of sitagliptin was reported between 1 and 4 h, and t_1/2_ was around 12 h. The fed state has a limited effect on its PK profiles. The T_max_ of metformin was reported between 7 and 10 h, and t_1/2_ was around 12 h. The state of fasting or fed was not revealed (FDA, 2005; FDA, 2006; FDA, 2012). The sampling point was designed to be quite dense within 12 h after dosing accordingly to depict accurate PK profiles of both drugs.

According to the JANUMET^®^ XR label (Janumet, 2012), hypoglycemia was one of the most frequently observed AEs in clinical trials. The FDA guidance suggested 60 mL of a 20% glucose solution in water administered every 15 min for up to 4 h after dosing in the *in vivo* study in case of hypoglycemia. Considering the risk of hypoglycemia in healthy subjects after taking one pill is relatively low, we did not follow the suggestion. In order to prevent the risk of hypoglycemia, we arranged blood glucose testing 2 and 8 h after dosing. The time point 2 h is close to the T_max_ of sitagliptin, and 8 h is around 4 h after lunch, both are the time points indicating a high possibility of hypoglycemia. We have also made a hypoglycemia emergency plan and lay in a supply of glucose water in case of hypoglycemia. In the fed study, none of the subjects experienced hypoglycemia. In the fasting study, one subject had transient hypoglycemia and recovered after taking 60 ml of a 20% glucose solution in water. The results demonstrated that our consideration on hypoglycemia prevention was practicable.

Most of the drug-related AEs reported in our study were known to date. According to JANUMET^®^ XR label (Janumet, 2012), a slight increase in the white blood cell count of patients treated with sitagliptin and metformin immediate-release compared to patients treated with placebo and metformin was observed. However, we observed one case of decreased white blood cell count in the fed and fasting study after the R drug administration, respectively. As the sample size is relatively small, we cannot conclude that the decrease in leukocyte count is a newly identified adverse reaction of JANUMET^®^ XR. More post-marketing experience is in need. In summary, the combination dose of sitagliptin and metformin were well tolerated in healthy subjects, the safety results were in line with previous reports (Shi et al., 2022; Schnaars et al., 2023).

As a selective DPPIV inhibitor, sitagliptin stabilizes and increases active incretins, namely glucose-dependent insulinotropic polypeptide (GIP) and glucagon-like peptide-1 (GLP-1) (Miller and St Onge, 2006), and metformin administration resulted in an increased total GLP-1 level (Mannucci et al., 2001). The effect of metformin on active GLP-1was complementary to those of sitagliptin. Given its limited hepatic metabolism and low protein binding, sitagliptin has a low potential for drug-drug interactions. No clinically significant drug-drug interactions were found between sitagliptin and metformin (Januvia, 2006; European Medicines Agency, 2021). The above facts indicated them as prime candidates for combination therapy.

Sitagliptin provided better efficacy than a placebo add-on to metformin and was non-inferior to glimepiride, glipizide, saxagliptin, and empaglifozin in improvements in HbA_1c_ levels (Charbonnel et al., 2006; Nauck et al., 2007; Scheen et al., 2010; Arechavaleta et al., 2011; Ahmed et al., 2022b). In addition, sitagliptin is generally well tolerated with a low risk of hypoglycaemia (Hanefeld et al., 2007; Janumet, 2022) and a neutral effect on bodyweight (Umezawa et al., 2015), thus lowering the risk of cardiovascular disease. On the contrary, sulfonylureas, meglitinides, thiazolidinediones, and insulin therapies all increase the risk of hypoglycaemia and bodyweight gain (Garber et al., 2015). GLP-1 RAs are more effective in terms of HbA_1c_ and body weight control than sitagliptin (Li et al., 2017). However, they require subcutaneous administration, usually cause severe gastrointestinal side effects, and are more expensive than sitagliptin (Nauck, 2016). It is noteworthy that a recent case-control cohort study found metformin plus sitagliptin might exert a protection against COVID-19 in T2DM patients, as evidenced by the mitigation of oxidative stress, CT scan score, and clinical outcomes (Al-Kuraishy et al., 2022). To sum up, with its convenient once-daily oral regimen, low potential for pharmacokinetic drug-drug interactions, and good efficacy and safety profiles, sitagliptin remains a critical add-on therapy to metformin in the management of T2DM patients (Karki et al., 2022). The fixed formulation of sitagliptin and metformin (extended-release) made a convenient once-daily oral regimen for T2DM patients. Sitagliptin 50 mg BID was equally effective to 100 mg QD in glycemic control. Lower doses were less effective, and doses up to 200 mg did not show an efficacy advantage (Aschner et al., 2006; Raz et al., 2006; Scott et al., 2007). Thus the 100 mg/1000 mg strength selected in our studies was prompt to be a popular dose regime.

Though patients can benefit from JANUMET^®^ XR, a relatively higher cost may render the prescribing decisions. It has been reported that sitagliptin was less cost-effective compared to empagliflozin as a second-line treatment to metformin (Reifsnider et al., 2021). Up to now, no generic form of JANUMET^®^ XR has been approved by FDA nor MHRA. The sitagliptin 100 mg and metformin 1000 mg produced by Nanjing Chia-Tai Tianqing Pharmaceutical Company was the first generic form of JANUMET^®^ XR approved by the National Medical Products Administration (NMPA) of China, which can make the medications more affordable to those patients who cannot afford brand formulations.

## 5 Conclusion

In summary, the present study was the first to study the bioequivalence of sitagliptin 100 mg and metformin 1000 mg produced by Nanjing Chia-Tai Tianqing Pharmaceutical Company to Janumet^®^ XR. The protocol design followed the FDA guidance with small modifications on the glucose water supplement. The results demonstrate both bio-equivalence and satisfactory safety of the T and R drugs. Our study may make a contribution to the drug accessibility of T2DM patients.

## Data Availability

The raw data supporting the conclusion of this article will be made available by the authors, without undue reservation.
